# Sonic hedgehog signalling mediates astrocyte crosstalk with neurons to confer neuroprotection

**DOI:** 10.1111/jnc.14064

**Published:** 2017-06-20

**Authors:** Christopher I. Ugbode, Imogen Smith, Benjamin J. Whalley, Warren D. Hirst, Marcus Rattray

**Affiliations:** ^1^ School of Pharmacy University of Bradford Bradford UK; ^2^ School of Chemistry, Food & Pharmacy University of Reading Reading UK; ^3^ Department of Biology University of York Heslington UK; ^4^ Portsmouth Brain Tumour Research Centre University of Portsmouth Portsmouth UK; ^5^ Neurodegeneration and Neurologic Diseases Pfizer Neuroscience Research Unit Cambridge Massachusetts USA; ^6^ Present address: Neurology Research Biogen 115 Broadway Cambridge MA 02142 USA

**Keywords:** cell culture, Gli1, multielectrode array, neurodegeneration

## Abstract

Sonic hedgehog (SHH) is a glycoprotein associated with development that is also expressed in the adult CNS and released after brain injury. Since the SHH receptors patched homolog‐1 and Smoothened are highly expressed on astrocytes, we hypothesized that SHH regulates astrocyte function. Primary mouse cortical astrocytes derived from embryonic Swiss mouse cortices, were treated with two chemically distinct agonists of the SHH pathway, which caused astrocytes to elongate and proliferate. These changes are accompanied by decreases in the major astrocyte glutamate transporter‐1 and the astrocyte intermediate filament protein glial fibrillary acidic protein. Multisite electrophysiological recordings revealed that the SHH agonist, smoothened agonist suppressed neuronal firing in astrocyte‐neuron co‐cultures and this was abolished by the astrocyte metabolic inhibitor ethylfluoroacetate, revealing that SHH stimulation of metabolically active astrocytes influences neuronal firing. Using three‐dimensional co‐culture, MAP2 western blotting and immunohistochemistry, we show that SHH‐stimulated astrocytes protect neurons from kainate‐induced cell death. Altogether the results show that SHH regulation of astrocyte function represents an endogenous neuroprotective mechanism.

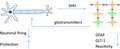

**Abbreviations used**
3D: 3 dimensional
anova: analysis of variance
ATP: adenosine triphosphate
CNS: central nervous system
DIV: days *in vitro*
E15: embryonic day 15
EAAT2: excitatory amino acid transporter 2
EFA: ethylfluoroacetate
FBS: foetal bovine serum
GFAP: glial fibrillary acidic protein
GLT‐1: glutamate transporter 1
Iba1: ionized calcium‐binding adapter molecule 1
Il‐1: interleukin 1
LPS: lipopolysaccharide
MAP2: microtubule‐associated protein 2
MAPK: mitogen‐activated protein kinase
MEA: multielectrode array
MTT: 3‐(4,5‐dimethylthiazol‐2‐yl)‐2,5‐diphenyltetrazolium bromide
NGS: normal goat serum
nNos: nitric oxide synthase
PAX6: paired box protein Pax‐6
PBS: phosphate‐buffered saline
PFA: paraformaldehyde
PTCH1: patched homolog 1
qPCR: quantitative polymerase chain reaction
SAG: smoothened agonist
SHH: Sonic hedgehog
SMO: smoothened receptor
SOX2: SRY (sex‐determining region Y)‐box 2

The hedgehog family of glycoproteins are signalling molecules with important roles in development and cell cycle regulation, with Sonic hedgehog (SHH) the only hedgehog protein expressed in the adult mammalian central nervous system (CNS) (Echelard *et al*. 1993; Traiffort *et al*. 1999). While the developmental implications of SHH signalling are well documented, only a few reports address the functional properties of SHH in the adult mammalian CNS.

SHH in the normal CNS is usually released by neurons (Sims *et al*. 2009; Gonzalez‐Reyes *et al*. 2012), with neuronal SHH able to reduce astrocyte reactivity (Chechneva *et al*. 2014; Chechneva and Deng 2015) and maintain the proliferative capacity of the neurogenic niche (Sirko *et al*. 2013). SHH responsiveness is present in astrocytes in normal and pathophysiological conditions as both components of the SHH membrane signalling complex, patched (PTCH1) and smoothened, are enriched in astrocytes (Cahoy *et al*. 2008; Zamanian *et al*. 2012) as is Gli1, a transcription factor essential for SHH signalling in the adult CNS (Garcia *et al*. 2010). Recent evidence using RNAseq identified Gli1/2/3 as astrocyte‐enriched transcription factors, localizing this pathway specifically to astrocytes (Zhang *et al*. 2014). Astrocytes are highly responsive to SHH (Atkinson *et al*. 2009; Yang *et al*. 2012; Sirko *et al*. 2013; Pitter *et al*. 2014). SHH stimulates proliferation of astrocytes as well as neural progenitor cells and olig2+ cells (Lai *et al*. 2003; Amankulor *et al*. 2009; Bambakidis *et al*. 2009; Sims *et al*. 2009). SHH released by neurons or astrocytes may act as a homeostat, regulating the phenotype of astrocytes, and this concept has been confirmed recently in the adult CNS (Farmer *et al*. 2016). Astrocytes can themselves secrete SHH after CNS injury (Yang *et al*. 2012; Pitter *et al*. 2014) and since astrocytes are SHH responsive, this signal is likely to act in a paracrine manner. Thus, SHH is placed to be a key factor that is involved in neuronal‐astrocyte signalling and regulation of astrocyte phenotype and function. The functional properties of astrocytes change depending on their state (Sofroniew 2009). Under normal non‐pathological circumstances, astrocytes possess a number of important functions including glutathione synthesis and efficient glutamate uptake via the glutamate transporter (GLT‐1) excitatory amino acid transporter 2 (Damier *et al*. 1993; Sofroniew and Vinters 2010). Under pathological conditions, astrocytes change their morphology contributing to neurodegeneration through increasing the levels of pro‐inflammatory cytokines and decreasing the levels of glutathione (John *et al*. 2004). This change in functional characteristics is described as a ‘reactive’ phenotype. The ‘reactive’ process involves a gradated change in cellular function; for example, the levels of glial fibrillary acidic protein (GFAP), a protein expressed predominantly by astrocytes, increases as astrocytes become more reactive giving insight to the level of reactivity (Middeldorp and Hol 2011). The idea of astrocyte ‘reactivity’ is changing, however, with recent research showing vast genetic heterogeneity between different types of injury; suggesting that classical markers such as shape change or GFAP immunofluorescence may be superseded by distinct patterns of gene transcription, specific to the type of injury (Zamanian *et al*. 2012). Indeed astrocytes have recently been classified as A1 or A2 based on their transcriptomic profiles after two distinct CNS injuries (Liddelow *et al*., 2017). A1 astrocytes are induced through microglial secretion of interleukin 1, tumour necrosis factor and C1q. These astrocytes become reactive and lose their ability to support neurons. A2 astrocytes, however, were thought to be protective as a result of their up‐regulation of neurotrophic factors after intraperitoneal lipopolysaccharide injection. This may help to disseminate the functional changes between normal and reactive astrocytes identifying whether reactive astrocytes gain ‘function’ or gain ‘detrimental effects’ dependant on the type of pathology (Sofroniew and Vinters 2010).

SHH signalling has been shown to contribute to both l‐glutamate and ATP release in cultured cerebellar astrocytes, identifying the importance of SHH in neuro‐glial interactions (Okuda *et al*. 2016). SHH has been reported to be neuroprotective in animal models of Parkinson's Disease (Miao *et al*. 1997; Dass *et al*. 2002; Hurtado‐Lorenzo *et al*. 2004). The protective properties of SHH have been described in the context of ischaemia (stroke), where small molecule agonists of this pathway have been shown to ameliorate some of the pathological features (Dellovade *et al*. 2006). Evidence now shows that stimulating the SHH pathway in mice days after induction of stroke, promotes repair, reduces infarct size and reduces reactive astrogliosis, namely astrocyte hypertrophy and increased deposition of GFAP (Chechneva *et al*. 2014). Recent evidence has shown that after glutamate induced excitotoxicity, nNos translocates to the nucleus and induces SHH transcription via Sox2 (Zhang *et al*. 2016), suggesting a specific link between SHH effects on astrocytes and neuroprotection.

Given that SHH reduces astrocyte reactivity *in vivo*, our aim was to understand what effect SHH agonists have on astrocyte morphology and on two markers of mature astrocytes, GFAP and GLT‐1. Furthermore, we aimed to investigate the link between SHH signalling, astrocyte reactivity and neuronal function using well‐established *in vitro* model systems and multielectrode array. Our data show SHH signalling transforms astrocytes, and potentiates a biochemically protective phenotype that suppresses neuronal hyperexcitability and confers neuroprotection against an excitotoxic insult.

## Materials and methods

### Animal groups

Timed mated female Swiss mice (Harlan UK) (RRID:IMSR_CRL:24) were maintained and killed in accordance with the UK Animals (Scientific Procedures) Act (1986). Animals were killed using cervical dislocation, according to Home Office guidelines. Cerebral cortices from embryonic day 15 mouse embryos were obtained and cells were isolated via mechanical disassociation as previously described (Ugbode *et al*. 2014).

### Cell culture and multielectrode array

Conventional (two‐dimensional) primary cortical astrocyte, cortical neuron and cortical astrocyte‐neuron co‐cultures were prepared as previously described (Bahia *et al*. 2012; Ugbode *et al*. 2014). For three‐dimensional (3D) transwell experiments, primary cortical astrocytes were seeded on Alvetex^®^ 6‐ and 12‐well hanging inserts (Reinnervate, Durham, UK) as previously described (Ugbode *et al*. 2016). The primary astrocyte cultures were of 95% purity. The remaining 5% consist of microglia and immature neurons. The astrocyte‐neuron cultures consist of 55% astrocytes to 40% neurons. The remaining 5% consist of microglia. Primary neuron cultures contained 90% neurons to 10% astrocytes. This is based on staining using CD68/ionized calcium‐binding adapter molecule 1 for microglia, GFAP/S100β for astrocytes, β‐3 tubulin/Tau/microtubule‐associated protein 2 (MAP2)/NeuN/Doublecortin staining for neurons (Data not shown).

For electrophysiological studies, primary neuron and astrocyte‐neuron co‐cultures were seeded on 60 channel (30 μm electrode diameter, 200 μm electrode spacing) planar multielectrode arrays (MEAs;60MEA100/10iR‐Ti‐gr – Multi Channel Systems, Reutlingen, Germany), as described previously (Hammond *et al*. 2013). MEAs were dried under UV light, coated in poly‐d‐lysine (0.1 mg/mL; Merck‐Millipore, Billerica, MA, USA) for 5 min, dried again and incubated overnight in 10% foetal bovine serum. Cells were plated at a density of 1 × 10^6^ per MEA and 50% media were exchanged every 3 days. At DIV 18, extracellular electrophysiological recordings (at least 300 s) were made from these cultures under control and drug‐treated conditions. For concentration‐response experiments, an equilibration period of 200 s following drug application was observed between any treatment and the start of the recording. Astrocytes were metabolically inhibited using ethylfluoroacetate (EFA; 1 mM; 1 h; Sigma, St Louis, MO, USA – Cat No: 163813), a derivative of fluoroacetate that acts as a metabolic toxin that prevents the conversion of citrate to isocitrate, inhibiting the tricarboxylic acid cycle (Clarke 1991).

MEA data were simultaneously recorded at 10 kHz/channel using MC_Rack (Multi‐Channel Systems, v4.3.5) and analysed by replay in the same software where raw data were high‐pass (200 Hz) filtered and spikes detected and re‐recorded using a threshold of −5.5 standard deviations from RMS noise. Spike trains were imported to NeuroExplorer software (Nex Technologies, Madison, AL, USA; version 4.088) where active electrodes (> 100 spikes recorded in a 300 s period in control conditions) were selected for further analysis. Spike rate histograms were constructed using NeuroExplorer for 300 s recordings in conditions examined and mean firing frequency for each electrode derived. Only data obtained from electrodes that were active through all conditions of the experiment were included in the analysis. The number of electrodes from each biological replicate was of a similar number (7–8 electrodes per experiment). Statistical analysis was done on pooled signals from each replicate experiment (i.e. *n* = 3).

### MTT assay

3‐(4,5‐dimethylthiazol‐2‐yl)‐2,5‐diphenyltetrazolium bromide (MTT) assay for cell viability was carried out as previously described (Ugbode *et al*. 2014). After treatment, primary mouse astrocytes were washed with HBM buffer and incubated with 500 μL of MTT buffer (0.5 mg/mL Thiazoyl Blue Tetrazolium Bromide (Sigma) in HBM) at 37°C for 1 h. The formazan precipitate was solubilized with 300 μL dimethylsulfoxide per well; 200 μL from each well was transferred to a 96‐well plate and absorbance measured using a plate reader (Flexstation 3; Molecular Devices, Palo Alto, CA, USA, λ = 490 nm).

### Immunofluorescence and cell imaging

Cells were treated with different compounds, washed and fixed for 30 min at room temperature (22°C) with 4% paraformaldehyde (Sigma) dissolved in phosphate‐buffered saline (PBS). After fixation, cells were blocked and permeabilized in 1% normal goat serum (NGS; Sigma) and 0.2% Triton‐X (Sigma) in PBS, for 1 h at room temperature (22°C). We used a polyclonal rabbit anti‐mouse GFAP primary antibody (Z0334; Dako, Carpinteria, CA, USA, UK, 0.6 μg/mL) or a monoclonal anti‐mouse MAP‐2 antibody (Cat no: 836201; Biolegend, San Diego, CA, USA. 1 : 1000) in PBS containing 1% NGS (PBS/NGS), allowing 1 mL per coverslip (12‐well plate) incubated overnight at 4°C.

Corresponding secondary antibodies were added (goat anti‐rabbit/mouse Alexafluors 488 and 568 in PBS/NGS, 2 μg/mL) for immunofluorescence (Life Technologies, Grand Island, NY, USA) incubating for 90 min, at room temperature 22°C. Cells were then incubated with Hoescht 33342 (4 μg/mL; Life Technologies), washed with PBS and mounted using Vectashield mounting medium (Vector Laboratories, Burlingame, CA, USA).

To assay cell proliferation, cells were incubated with anti‐Ki‐67 antibody (primary polyclonal rabbit anti‐mouse, 1 : 1000; Abcam ab66155, Cambridge, UK) along with a goat anti‐rabbit secondary conjugated to Alexafluor 488 following the above method. Antigen retrieval was carried out by incubation with citric acid (pH 6.0, 0.01M) for 1 h at 37°C before adding the primary antibody. To monitor proliferation, six images were analysed per condition (coverslips) across four independent experiments.

Images were collected on an (Zeiss Axioskop 2, Oberkochen, Germany) upright microscope using 10×/20× and 40× Plan‐NeoFluar objectives, using Zeiss Filter Sets 02/10 and 15 for 4,6‐Diamidino‐2‐phenylindole, dihydrochloride (DAPI)/FITC/Rhodamine. Images were processed using Axiovision Software (Zeiss) and quantitative measurements performed using ImageJ (1.43 u) (Image J Bethesda MD, USA). High resolution images were prepared using Photoshop CS3.

The resource identifiers for the primary and secondary antibodies are as follows: Primary Antibodies GFAP: Dako Cat# Z0334 RRID:AB_10013382; MAP2: Covance Research Products Inc Cat# SMI‐52R‐100 RRID:AB_510028, Bethesda, MD, USA; Ki‐67: Abcam Cat# ab66155 RRID:AB_1140752; GAPDH: Thermo Fisher Scientific Cat# AM4300 RRID:AB_2536381, Waltham, MA, USA, Waltham, MA, USA; Secondary Antibodes: Goat anti‐Mouse 488 Alexa Fluor: Thermo Fisher Scientific Cat# A‐11001 RRID:AB_2534069; Goat anti‐Mouse 546 Alexa Fluor: Thermo Fisher Scientific Cat# A‐11003 RRID:AB_2534071; Goat anti‐Rabbit 488 Alexa Fluor: Thermo Fisher Scientific Cat# A‐11034 also A11034 RRID:AB_2576217; Goat anti‐Rabbit 546 Alexa Fluor: Thermo Fisher Scientific Cat# A‐11010 RRID:AB_2534077.

### Time lapse microscopy and morphometric analysis

For time lapse microscopy, we used a Nikon ECLIPSE TE200 upright time lapse microscope and complementary Nikon NIS‐Elements (4.0) software, Kingson Upon Thames, UK was used to assay morphological changes. After cell treatment, the plate was placed in a heated chamber (37°C and 5% CO_2_) and fixed to the microscope platform. Images were taken once every 10 min for 24 h, generating 144 TIFF files. All collected images from an individual well formed an ND2 file, which was then opened in ImageJ, and saved as an AVI file allowing video playback. Individual frames were assembled into figures using Photoshop CS3. At the end of the time lapse, the cells were removed from the heated chamber, washed and fixed for further immunohistochemical investigation.

The NeuronJ plugin for ImageJ was used to measure the length of astrocytes. Briefly, TIFF files generated as mentioned above and individual images at specific time points (every 4 h from 0 to 24 h) were thresholded and astrocyte length calculated. Values were combined and average astrocyte length calculated. Twenty astrocytes were measured at each time point and three videos per treatment were analysed (Figure S1).

### Western blotting

Western blotting was carried out as previously described (Ugbode *et al*. 2014). Primary antibodies for GFAP (1 : 5000), GLT‐1 (monoclonal rabbit anti‐mouse – 1 : 10 000 – provided by Prof. David Pow, RMIT University), MAP2 (monoclonal anti‐mouse 1 : 1000; Biolegend, San Diego, CA, USA – 836201) and GAPDH (monoclonal goat anti‐mouse – 1 : 10 000; Life Technologies) were incubated overnight at 4°C in Tween/Tris buffered salt solution (TTBS) (20 mM tris buffer (pH 7.5; Sigma) containing 0.4% Tween‐20 (Sigma) and 1% non‐fat milk powder). Goat antirabbit/goat antimouse secondary antibodies conjugated to horseradish peroxidase (Sigma) were used at 1 : 5000 prior to detection with enhanced chemiluminescence reagent (Clarity; Biorad, Hemel Hempstead, UK). Molecular weight markers (Biorad Prestained Dual Xtra Standards) were run alongside lysates and GAPDH protein levels were used as a loading control. Blots were imaged using the Chemidoc MP imaging system (Biorad) for chemiluminescence and analysed by the corresponding ImageLab software (Biorad). Images were exported from the software at a resolution of 600 dpi into photoshop CS3 and processed into figures.

### Quantitative PCR

RNA was extracted using RNA Bee (AMS Biotechnology, Abingdon, UK) according to manufacturer instructions. All RNA samples were derived from cells growing in six‐well plates (8 × 10^5^cells per well). RNA concentrations were calculated using a NanoDrop 2000 spectrophotometer (Thermo Fischer Scientific). A high‐capacity cDNA kit (4368814; Life Technologies) was used to transcribe 2 μg of RNA. To perform qPCR, cDNA was diluted 1 : 20 in Tris‐EDTA buffer (Sigma) and 5 μL of cDNA was combined with 1 μL of both the forward and reverse primers (2.5 μM) and 7 μL of SYBR green master mix (cat no: 204141 – QuantiTect SYBR Green PCR Kit; Qiagen, Valencia, CA, USA) to create a 14 μL reaction volume per well. Oligonucleotide sequences were created using Primer3 (Koressaar and Remm, 2007) and PUBMED websites and custom synthesized (Sigma). Primers were re‐suspended in nuclease‐free water, to a concentration of 2.5 μM. All primers were validated using a primer efficiency test before use. All oligonucleotides here have an efficiency ≥ 90%. Primer sequences for Gli‐1 (ID number: 90186272b1), Gli‐2 (124487480b1), Gli‐3 (120953172b1), GLT‐1 (227330634c1) and GAPDH (12601253861) were obtained from PrimerBank (Spandidos *et al*. 2010) and oligonucleotides for amplification of GFAP and PTCH1 were designed from the published sequences as follows, GFAP: (F) 5′‐GGTGAGCCTGTATTGGGACA‐3′ (bases) and (R) 5′‐TTTCTCCAACCTCCAGATCC‐3′ (bases 1223 – 1335, fragment size 112 base pairs); PTCH1: (F) 5′‐CACAGCAACAGTCACCGAA‐3′ (bases 3012‐3032) and (R) 5′‐GTTCTCACAACCCTCGGAAC‐3′ (bases 3992 – 4101, fragment size 118 base pairs).

### Aconitase assay

To assay the activity of mitochondrial aconitase, we used an aconitase activity kit (ab83459; Abcam), in accordance with the manufacturer's instructions. Primary astrocytes and neurons in six‐well plates were treated with 200 μL media or 200 μL of ethylfluoroacetate EFA for 1 h. After treatment, cells were homogenized in 100 μL of assay buffer provided and spun at 20,000 g for 15 min at 4°C, to separate the mitochondrial fractions. The pellet was re‐suspended with 100 μL of assay buffer and sonicated for 20 s. Samples were then incubated for 1 h with the substrate mixture at 25°C. Afterwards, 10 μL of developer was added to each sample, incubated at 25°C for 10 min and the absorbance was measured using a plate reader (Flexstation 3; Molecular Devices. λ = 450 nm). Absorbance was then compared to an isocitrate standard curve.

### MAP2 assay

MAP2 protein levels were used to assay kainate‐induced neurodegeneration. To assay the effect of SHH‐sensitive astrocytes on neurons, we used 3D Alvetex scaffolds to culture primary astrocytes. The astrocytes were seeded into the scaffold and after 11–12 DIV, the 3D well inserts were treated with smoothened agonist (SAG) (1 μM) for 4 h and along with untreated 3D scaffolds, were washed twice with PBS and then incubated with cultures of primary neurons (14–18 DIV) for 24 h. After 24 h, inserts were removed and the neurons were incubated with 100 μM kainic acid for 24 h. Cells were either fixed in paraformaldehyde and used in immunohistochemistry or lysed and used for western blotting. Images obtained after MAP2 staining were thresholded and quantified using Volocity software (Edition 6.2; Perkin Elmer, Waltham, MA. USA).

### Drug concentrations

Concentration‐response experiments for SAG/purmorphamine (Pur)/Cyclopamine were carried out using time lapse microscopy (data not shown). Furthermore, drug toxicity was assayed in mixed cultures using multielectrode array. Our time course experiments were based on previously published *in vitro* experiments on cultured neurospheres (Sirko *et al*. 2013) and astrocytes (Atkinson *et al*. 2009). Kainic acid‐induced neurodegeneration has been previously characterized in our laboratory and chosen concentrations were informed by published literature (Wang *et al*. 2005).

### Statistical analyses

Statistical significance was derived using one‐way anova followed by Dunnett's multiple comparison *post hoc* test unless otherwise stated (Prism 6; GraphPad Software, San Diego, CA, USA).

## Results

### Sonic hedgehog pathway activation causes elongation and proliferation of primary mouse astrocytes

**Figure 1:**
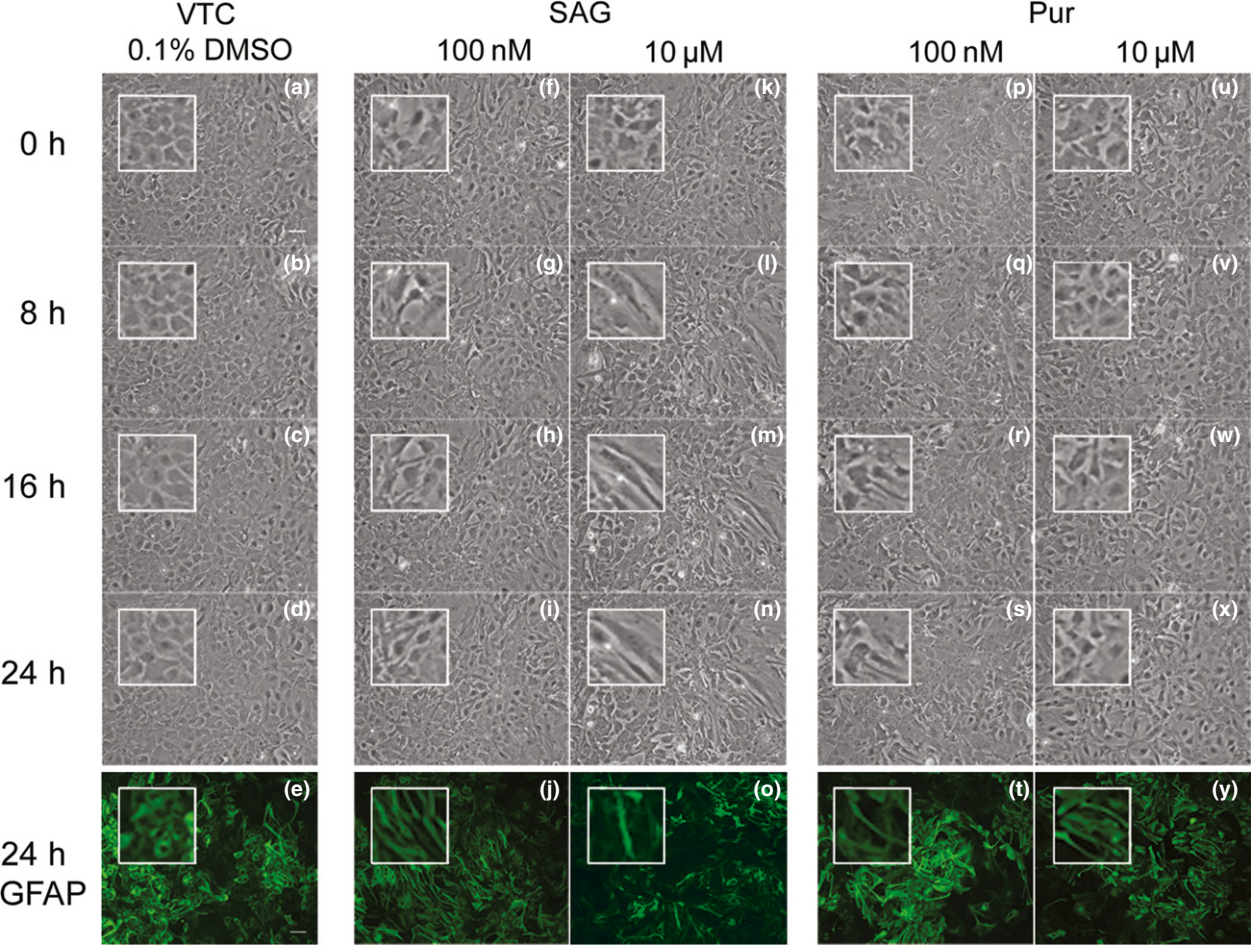
Sonic hedgehog (SHH) pathway agonists cause astrocyte elongation over time. Phase contrast time lapse microscopy identifies morphological changes in primary mouse astrocytes (embryonic day 15, 12 DIV) treated with SHH agonists, Smoothened agonist (SAG) and Pur (purmorphamine). Astrocytes were visualized following vehicle administration (a–d), or the SHH agonists SAG (f–i and k–n) or Pur (p–s and u–x). Cultures were also fixed and immunostained for glial fibrillary acidic protein (GFAP) (Green e, j, o, t, y). Over 24 h, astrocytes begin to elongate showing morphological changes indicative of a transition to a less reactive (de‐differentiated) state. Note the change from cobblestone shape to more elongated morphologies. White boxes shows zoomed cells, highlighting change in morphology of those cells over time. The phenotypic change was evident between 4 and 8 h for purmorphamine and within 1 h for SAG. Scale bar = 10 μm.

To analyse SHH pathway stimulation in primary astrocytes, we used two chemically distinct agonists, Pur and SAG, to induce pathway stimulation in primary astrocytes through the smoothened receptor (Chen *et al*. 2002; Sinha and Chen 2006). Time lapse and fluorescence microscopy showed that Pur and SAG caused astrocytes to elongate (Fig. 1). Both agonists displayed different time and concentration dependence, with SAG (elongation after 1 h) acting more rapidly than Pur (4–12 h). Furthermore, at 10 μM, SAG induced a persistent elongation over 24 h in comparison to Pur (Figure S1). In contrast, no obvious changes to morphology were observed under treatments with the SHH pathway antagonist, cyclopamine (data not shown).

### SHH agonists cause cell proliferation

**Figure 2:**
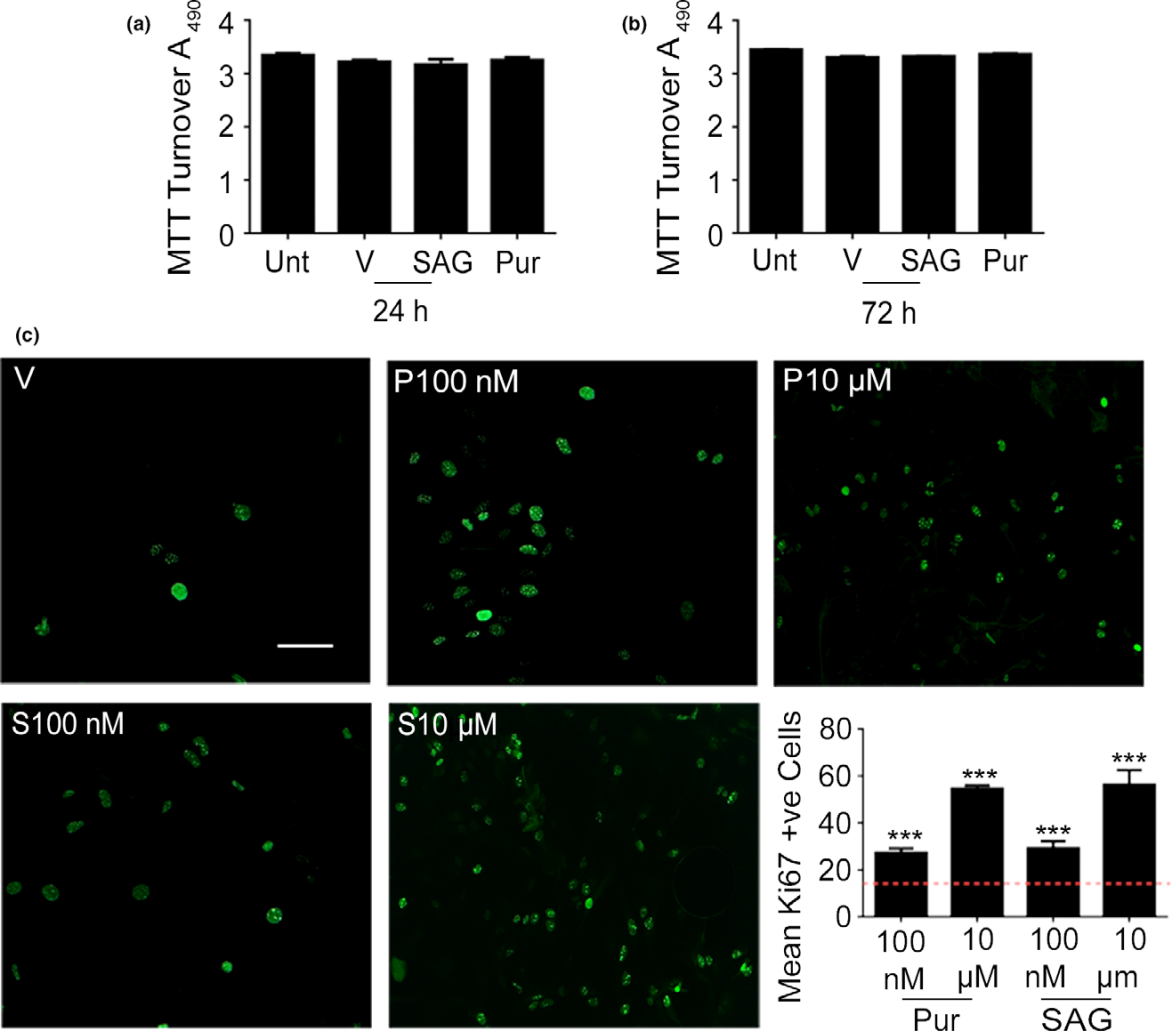
Sonic hedgehog pathway stimulation causes astrocyte proliferation. Smoothened agonist (SAG) and purmorphamine (Pur – both 10 μM) do not change 3‐(4,5‐dimethylthiazol‐2‐yl)‐2,5‐diphenyltetrazolium bromide (MTT) turnover at 24 (a) or 72 (b) hours in primary mouse astrocyte cultures (embryonic day 15, 12 DIV). Immunostaining (c) for Ki67 (green), a marker of cell division reveals increased astrocyte proliferation with both agonists. Scale bar = 20 μm. Statistical analysis was carried out using one‐way analysis of variance (anova) followed by Dunnett's *post hoc* test (*p<0.05, **p<0.01, ***p < 0.001). Error bars represent standard error of the mean (SEM). *N* = 3 independent cultures.

Next, we monitored astrocyte viability and proliferation following SHH pathway stimulation. We first used MTT turnover as a biochemical readout of cellular viability. Using analysis of variance (anova,* F* = 1.4, *p* = 0.31) followed by Dunnett's *post hoc* test, no significant differences were observed between SAG‐ and Pur‐treated cells (10 μM) when compared to untreated controls (Fig. 2a and b) at either 24 (SAG; 3.2 ± 0.08, *p* = 0.1762; Pur; 3.3 ± 0.05, *p* = 0.5982, DF = 8, *n* = 3) or 72 h (SAG; 3.3 ± 0.1, *p* = 0.565; Pur; 3.3 ± 0.1, *p* = 0.0535, DF = 8, *n* = 3), supporting evidence from time lapse microscopy that shows no cellular toxicity following agonist exposure.

Since the SHH pathway is known to be mitogenic, we used anti‐Ki‐67 antibodies to monitor astrocyte proliferation following treatment with the SHH agonists (Fig. 2c). We observed a significant increase (*F* = 32, DF = 86, *p* = 0.0001) compared to vehicle‐treated controls in the number of dividing cells per field under both SAG (100 nM; 29.3 ± 3 cells, *p* < 0.0001 and 10 μM; 56.3 ± 5.9 cells, *p* < 0.0001, *n* = 4) and Pur (100 nM; 27.4 ± 1.8 cells, *p* = 0.0005 and 10 μM; 54.7 ± 1.2 cells, *p* < 0.0001, *n* = 4) as analysed by one‐way anova followed by Dunnett's *post hoc* test.

**Figure 3:**
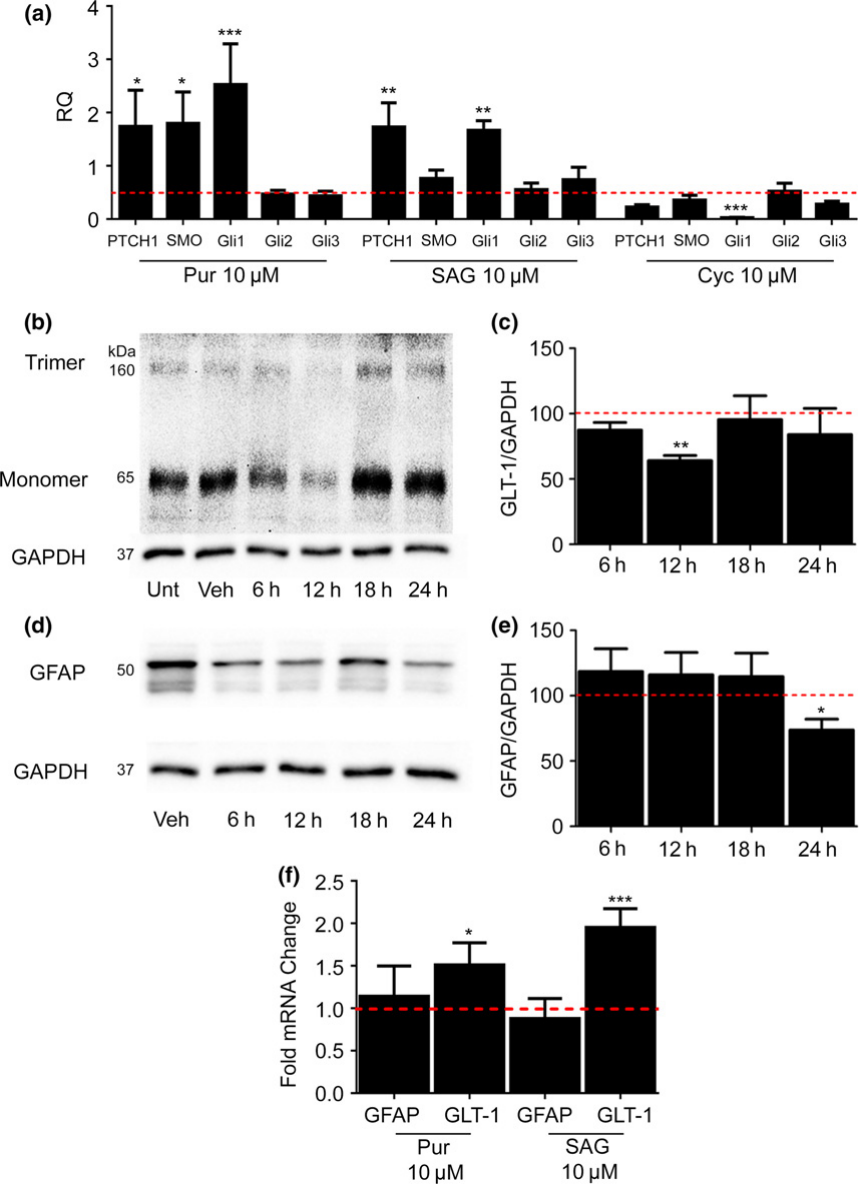
Sonic hedgehog pathway stimulation increases Gli1 mRNA and decreases astrocyte hallmarks glutamate transporter 1 (GLT‐1) and glial fibrillary acidic protein (GFAP). qPCR and western blotting was carried out for a number of mature astrocyte markers using cDNA and protein samples from murine embryonic (embryonic day 15) cortical astrocytes (12 DIV). (a) qPCR shows both agonists significantly increase Gli1 mRNA with smoothened agonist (SAG) further increasing patched homolog 1 (PTCH1) and smoothened receptor (SMO) mRNA. Pur (Purmorphamine, 10 μM) also significantly increases PTCH1 mRNA. The antagonist cyclopamine significantly reduces Gli1 mRNA. Panel b shows GLT‐1 (trimer 160 kDa and monomer 65 kDa) protein levels after time course treatments with SAG (10 μM). GLT‐1 protein levels significantly decrease after 12 h treatment with SAG. Panel d shows (GFAP, 50 kDa) protein levels after time course SAG (10 μM) treatments. GFAP protein levels are significantly reduced after 24 h SAG treatment. Glyceraldehyde Phosphate Dehydrogenase (GAPDH) was used as a sample loading control. GLT‐1 (c) and GFAP (e) protein levels were quantified by normalizing to GAPDH. Protein levels after treatment are expressed as a % of the untreated control protein levels (red line). Statistical analysis was carried out using anova (analysis of variance). (f) qPCR shows 24 h treatments with either SAG (10 μM) or Pur (10 μM) significantly increase GLT‐1 mRNA, while GFAP mRNA remains unchanged. mRNA changes analysed through relative quantification (RQ) and are depicted as the mean compared to untreated controls (red line). GAPDH was used as a reference gene during relative quantification of CT values. Statistical analysis was carried out using anova followed by Dunnett's *post hoc* test (*p < 0.05, **p < 0.01, ***p < 0.001). Error bars represent standard error of the mean (SEM). *N* = 5 biological replicates for both western blotting and qPCR data.

To confirm that agonists were acting on the SHH signalling pathway, we carried out qPCR to measure the mRNA levels of the main transcription factor recruited following Smo signalling, Gli1, along with other transcripts associated with hedgehog signalling. Stimulating the SHH pathway revealed significantly increased Gli1 mRNA following 24 h treatments with Pur (5.4 ± 1.8‐fold increase, *F* = 5.6, *p* = 0.0010, DF = 56, *n* = 6) and SAG (2.3 ± 0.03‐fold increase, *F* = 5.8, DF = 41, *p* = 0.0022, *n* = 6). Pur and SAG did not change Gli2 or Gli3 mRNAs. Levels of mRNA for PTCH1, the transmembrane receptor which inhibits Smo activation, increases after both Pur (4.1‐fold ± 1.9, *F* = 5.6, *p* = 0.0352, DF = 56, *n* = 6) and SAG (2.5‐fold ± 0.8 increase, *F* = 5.8, *p* = 0.002, DF = 41, *n* = 6) treatments (Fig. 3a).

We also treated primary mouse astrocytes with cyclopamine, a SHH pathway antagonist (10 μM, 24 h) (Fig. 3a). Gli1 mRNA levels determined by qPCR were significantly reduced (0.94‐fold ± 0.01 decrease, *F* = 9.1, *p* = 0.0004, DF = 26, *n* = 5) as determined by one‐way anova followed by Dunnett's *post hoc* test. This result shows there is tonic SHH signalling in astrocyte cultures.

### SHH agonists down‐regulate GLT‐1 and GFAP protein levels

SHH pathway stimulation has previously been shown to transform astrocytes, correlating with a decrease in GFAP (Yang *et al*. 2012; Sirko *et al*. 2013); however, there are no reports on expression of the glutamate transporter, GLT‐1, an abundant CNS protein which is a marker of mature astrocytes. Using western blotting, we found SAG (10 μM) significantly reduced GLT‐1 after 12 h (42.6 ± 8.5% decrease, *F* = 6.4, *p* = 0.0085, DF = 12, *n* = 5) with levels returning to control levels at 24 h (Fig. 3b and c) as determined by one‐way anova followed by Dunnett's *post hoc* test. Purmorphamine (10 μM) also reduced GLT‐1, but the knockdown was only evident after 24 h (50.2 ± 9% decrease, *p* = 0.010, DF = 8, *n* = 5, Students *t*‐test; data not shown), consistent with the slower effect of Pur compared to SAG on astrocyte morphological changes. Both SAG and Pur caused significant decreases in the levels of GFAP (Fig. 3d and e) (*F* = 4.3, *p* = 0.03, anova). SAG (10 μM, 24 h) caused 28.7 ± 8.8% decrease compared to controls (*p* = 0.0325, DF = 17, *n* = 5), and Pur (10 μM, 24 h) caused 51 ± 9% decrease compared to controls (*F* = 7.4, *p* = 0.0027, DF = 8, *n* = 5).

In order to explore whether GFAP and GLT‐1 mRNA correlated with protein levels, we also assayed GFAP and GLT‐1 transcripts using qPCR (Fig. 3f). GFAP mRNA showed no statistical differences between control and treated cells, indicating that the loss of GFAP protein was post‐transcriptional. Significant increases in GLT‐1 mRNA were observed following treatment of astrocytes with Pur (0.51 ± 0.2‐fold increase *p* = 0.0322, DF = 11, *n* = 4) or SAG (0.95 ± 0.2‐fold increase, *p* = 0.0004, DF = 11, *n* = 4) after 24 h, suggesting that the increase in GLT‐1 protein between 12 and 24 h was caused by enhanced availability of GLT‐1 mRNA.

### Smoothened agonist signals via astrocytes to decrease neuronal firing

Having shown that activation of SHH signalling pathways caused changes in levels of key astrocyte proteins which accompanied the morphological changes, we addressed the functional consequences of SHH pathway activation. To test how SHH treatment of astrocytes affects neurons, we used MEA to allow simultaneous electrophysiological recordings from up to 60 electrodes onto which neurons are cultured (Hammond *et al*. 2013). We cultured primary cortical neurons and astrocyte‐neuron co‐cultures and observed the effect of pathway stimulation upon neuronal firing frequency.

**Figure 4:**
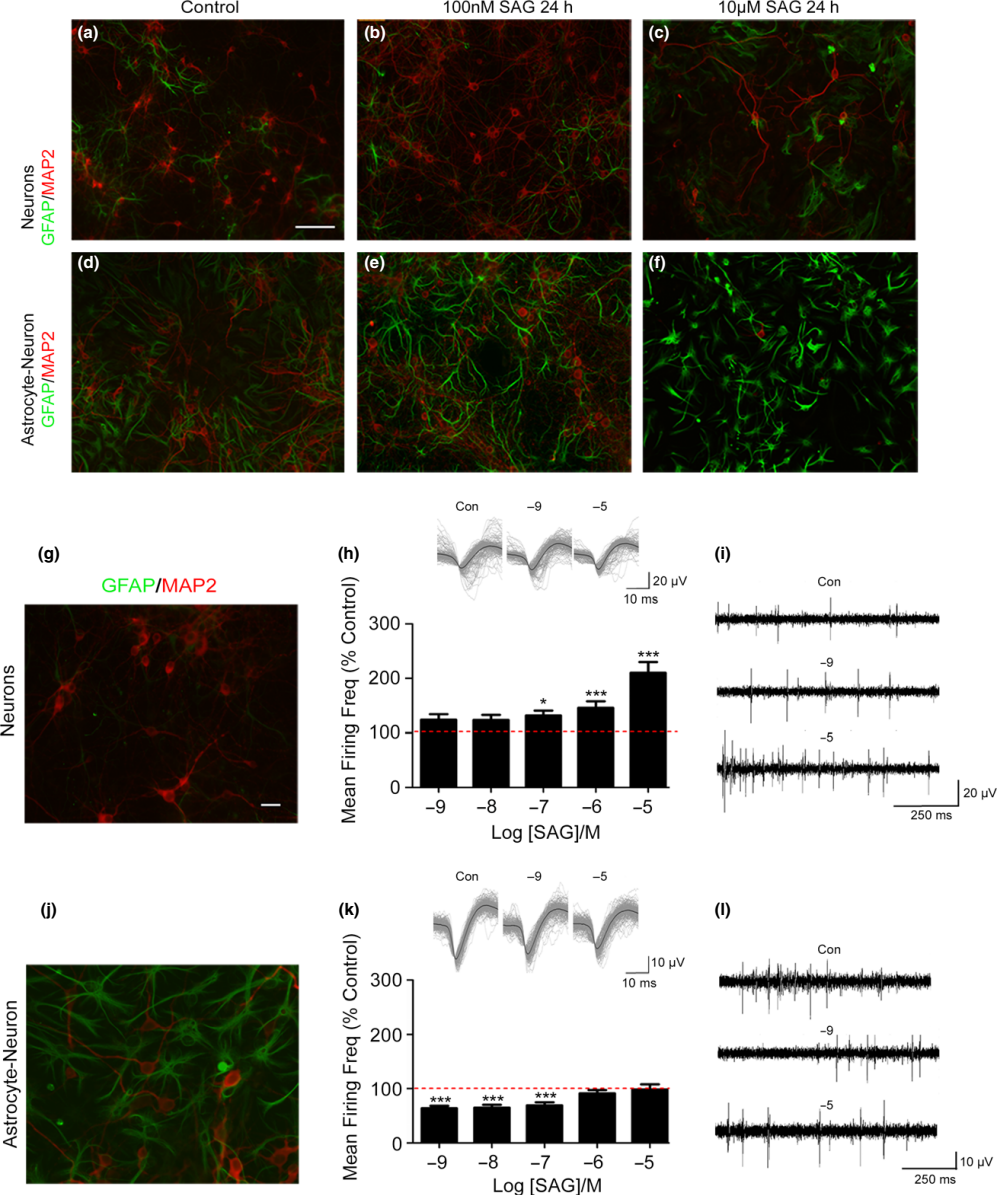
Smoothened agonist acts through astrocytes to reduce neuronal firing frequency. Immunohistochemistry showing glial fibrillary acidic protein (GFAP – green) and MAP2 (microtubule‐associated protein 2 – red) expression in primary cortical neurons (a–c) or astrocyte‐neuron cultures (d–f). Low concentrations of smoothened agonist (SAG) do not visibly alter either culture (b and e). High concentrations of SAG (10 μM) cause neuronal cell death in both neurons (c) and astrocyte‐neuron cultures (f). Scale bar = 100 μm. Multielectrode array (MEA) recording of neuronal spike firing activity reveals that the effect of SAG upon neurons is mediated by astrocytes. In neuron only cultures (g) SAG increases neuronal firing in a concentration‐dependent manner (h). Insets show 200 spike overlay (light grey) and average spike waveform (white) in control, 10 nM and 10 μM conditions. Scale: 20 μV, 10 ms. Representative traces (i) demonstrate concentration‐dependent increases in spike firing. Scale: 20 μV. 250 ms. In astrocyte‐neuron cultures (j), astrocytes bind SAG which leads to a decrease in neuronal firing (k). Insets show 200 spike overlay (light grey) and average spike waveform (white) in control, 10 nM and 10 μM conditions. Scale: 10 μV, 10 ms. Higher concentrations (10 μM) cause firing frequency to increase back to 100% of control suggesting that astrocytes have limited capacity to bind the agonist. Representative traces (L) demonstrate concentration‐dependent decreases in spike firing. Scale: 10 μV. 250 ms. Scale bar = 10 μm. Significance was derived using analysis of variance (anova) followed by Dunnett's *post hoc* test (*p<0.05, **p<0.01, ***p < 0.001). Error bars represent standard error of the mean (SEM). *N* = 3 independent cultures. Data for each replicate culture is from 7 to 9 active electrodes.

To account for potential effects of neuronal toxicity on electrophysiological readouts, we generated timed matched cultures for immunohistochemistry. Using MAP2 staining, we found that low (100 nM, 24 h) concentrations of SAG have no effect on neuronal viability in neurons (Fig. 4b) alone or in astrocyte‐neuron co‐cultures (Fig. 4e), whereas high concentrations at long exposures (10 μM, 24 h) kill neurons in both neuronal cultures (Fig. 4c) and co‐cultures (Fig. 4f). This allowed the concentration range and exposure time to be chosen for MEA experiments. SAG was used for all electrophysiology experiments because of its solubility in saline as Pur is only soluble in dimethylsulfoxide, a vehicle that can affect neuronal firing in its own right in MEA experiments (data not shown).

In primary cortical neuron cultures (18 DIV), SAG (after 5 min incubation) produced a statistically significant concentration‐dependent increase in firing frequency (Fig. 4h). At 100 nM, SAG caused 30.3 ± 8.7% increase (*F* = 27.6, *p* = 0.0121, DF = 143 *n* = 3), at 1 μM a 49 ± 13.2% increase (*F* = 27.6, *p* < 0.0001, DF = 143, *n* = 3) and at 10 μM, a 103 ± 15.6% increase (*F* = 27.6, *p* < 0.0001, DF = 143, *n* = 3). Data represent 24 active electrodes across three biological replicates. Conversely, in co‐cultures containing astrocytes, SAG did not increase neuronal firing frequency (Fig. 4k). Here, neuronal firing frequency was significantly decreased compared to vehicle‐treated controls 5 min following 1 nM SAG (*F* = 16, 36.3 ± 4.2% decrease, *p* < 0.0001, DF = 167, *n* = 3), 10 nM (*F* = 16, 35.3 ± 5.2% decrease, *p* = 0.0001, DF = 167, *n* = 3) and 100 nM (*F* = 16, 31.2 ± 5.6% decrease, *p* = 0.0009, DF = 167, *n* = 3) concentrations of SAG. However, at higher concentrations of SAG, the previously detected significant decreases in neuronal firing frequency were no longer evident (1 μM; 8.8 ± 6% decrease, *p* = 0.7587, *n* = 3 and 10 μM; 1.9 ± 9.5% decrease, *p* = 0.9997, *n* = 3.). We note that, in support of our immunohistochemical data, the highest concentration of SAG (10 μM) when applied for 1 h or more, caused a large decrease in neuronal firing frequency in co‐cultures (10 μM SAG, 1 h; 63 ± 8.4% decrease compared to control, *F* = 16, *p* < 0.0001, DF = 167, *n* = 3) indicative of toxicity.

### Metabolic inhibition of astrocytes abolishes SHH signalling to neurons

That SAG caused elevated neuronal firing in neuron cultures, but not in astrocyte‐neuron co‐cultures could mean that astrocytes are binding SAG passively so that less agonist reaches neurons or that SAG binding to astrocytes causes them to actively suppress neuronal firing. In order to distinguish between these possibilities, we chose to metabolically inhibit astrocytes. We used EFA which is a selective metabolic inhibitor of astrocytes (Hassel *et al*. 1992).

**Figure 5:**
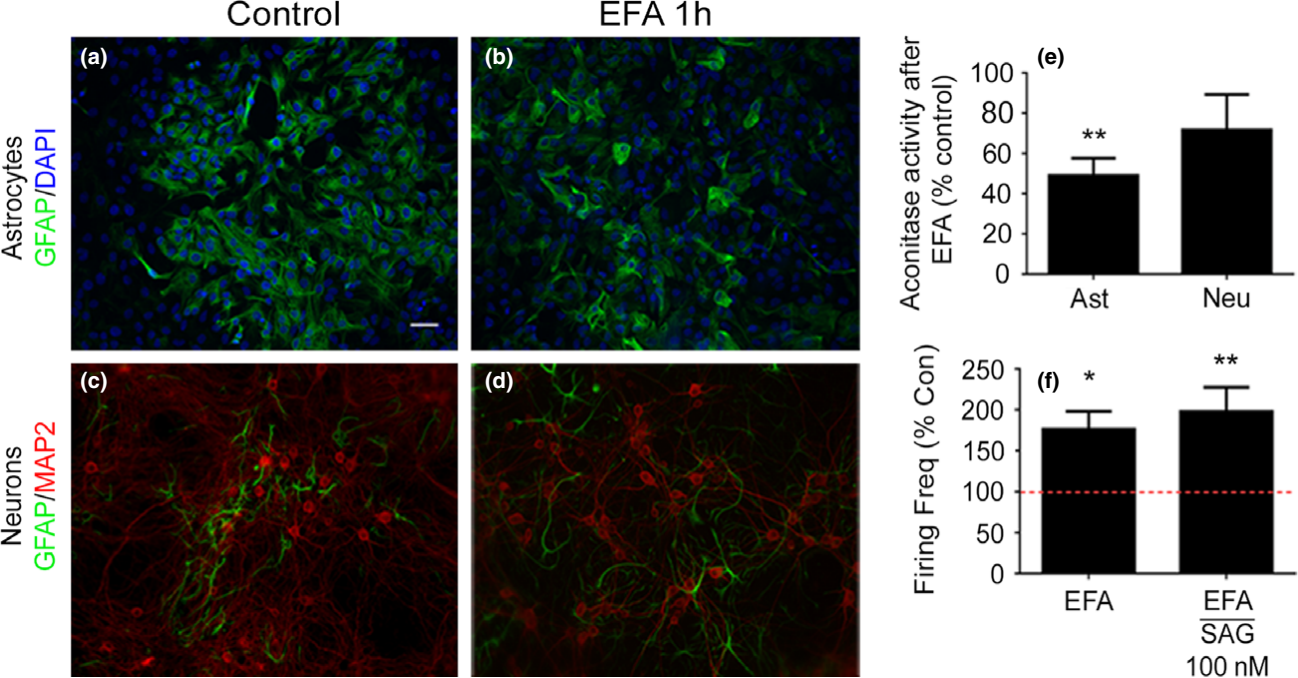
Neuronal firing properties in smoothened agonist (SAG)‐treated co‐cultures are dependent on metabolically active astrocytes. Immunohistochemistry showing glial fibrillary acidic protein (GFAP – green) expression in primary astrocytes (a and b) and MAP2 (microtubule‐associated protein 2 – red) expression in primary cortical neuron cultures (c and d). Ethylfluoroacetate (EFA, 1 mM, 1 h) does not visibly change astrocytes (b) or neuronal (d) cultures when compared to controls (a and c). Furthermore, EFA (1 mM, 1 h) significantly reduces mitochondrial aconitase activity in astrocytes (e). While EFA decreases aconitase activity in neurons, the observed decrease was not statistically significant. *N* = 5 independent cultures. Finally, multielectrode arrays (MEA's) were treated with EFA and then SAG (100 nM). EFA alone significantly increases firing frequency in co‐cultures. This increase in firing rate is further enhanced following immediate incubation with SAG (smoothened agonist, 100 nM) when compared to controls (red line). Statistical analysis carried out using one‐way anova followed by Dunnett's multiple comparison test (*p < 0.05, **p < 0.01)). Error bars represent standard error of the mean (SEM). *N* = 3. Scale bar = 10 μm. Data represent 56 active electrodes across three biological replicates.

Time matched cultures of primary astrocytes and primary neurons were prepared, treated with EFA (1 mM, 1 h) and stained for GFAP and MAP2. Primary astrocytes (Fig. 5b) and neurons (Fig. 5d) show no differences under EFA indicating that EFA does not cause cell death under these conditions. In order to demonstrate that astrocyte metabolism is preferentially inhibited under EFA, we applied EFA for 1 h (1 mM) to pure cultures of primary astrocytes and primary neurons and then assayed the activity of aconitase and compared the activity between cultures. We observed a statistically significant decrease in aconitase activity in astrocytes (51 ± 8.6% decrease, *p* = 0.0079, *n* = 4) but not in primary neurons (28 ± 18% decrease, *p* = 0.1534, *n* = 4), as determined by one‐way anova (Fig. 5e). This shows that astrocytic aconitase is preferentially inhibited by EFA over neuronal aconitase.

We then treated co‐cultures with EFA for 1 h and further treated the MEA's with 100 nM SAG or vehicle and measured neuronal firing frequency 5 min after drug application and then over the following hour. This experiment allowed us to test both the effect of EFA on firing frequency and also observe differences in firing frequency between EFA (1 h) and EFA and SAG (100 nM, 1 h). EFA alone causes a significant increase in firing frequency compared to controls (*F* = 6, 75 ± 22%, *p* = 0.0222, DF = 169, *n* = 3), indicating metabolic suppression of astrocytes causes some hyperexcitability. The addition of SAG to EFA‐treated astrocytes, rather than causing a suppression in firing frequency as previously shown, caused a statistically significant increase in firing frequency compared to controls (*F* = 6, 98 ± 29% increase, *p* = 0.0024, DF = 169, *n* = 3) as determined by one‐way anova followed by Dunnett's multiple comparison test (Fig. 5f).

### SHH sensitive astrocytes reduce kainate‐induced neurodegeneration

Since SAG treatment of astrocytes causes suppression of neuronal firing frequency, we wished to test whether this was associated with neuroprotection. We cultured primary astrocytes in Alvetex^®^ transwell inserts, allowing us to first stimulate astrocytes with SAG away from neurons, and following washout of SAG, allow the astrocytes to condition neuronal media without physical contact between astrocytes and neurons. We have previously characterized the properties of astrocytes cultured in 3D Alvetex^®^ transwell inserts (Ugbode *et al*., 2016). Primary astrocyte cultures (12 DIV) were left untreated, or treated with 1 μM SAG for 4 h. After treatments, the 3D transwells were washed twice with PBS to remove any SAG present in the astrocyte medium. The astrocyte transwells were then placed over wells containing primary neuronal cultures (14–18 DIV) for 24 h, to allow the astrocytes to condition the media. After 24 h, the transwells were removed and the neurons were incubated with 100 μM kainic acid for 24 h and neuronal viability was assayed using western blotting and immunohistochemistry for the mature neuron marker, MAP2.

**Figure 6:**
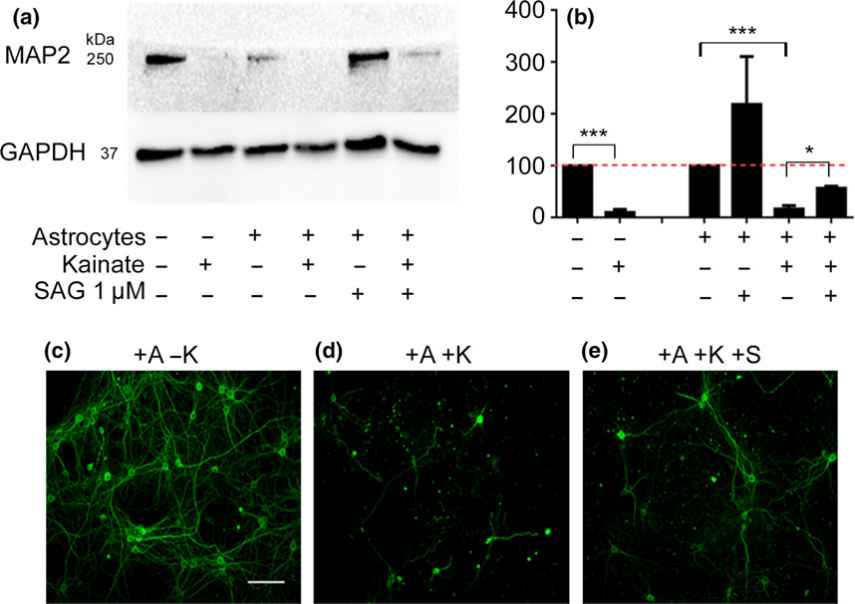
Sonic hedgehog (SHH)‐treated astrocytes delay kainate‐induced neurodegeneration *in vitro*. Western blotting was carried out to assay levels of MAP2 (microtubule‐associated protein 2) in protein samples derived from murine embryonic (embryonic day 15) cortical neurons (18 DIV). Kainate (100 μM, 24 h) induces significant neurodegeneration in neuronal cultures. Astrocytes co‐cultured with neurons using transwells, prior to addition of kainate, do not protect against kainate‐induced neuronal cell death. SHH‐treated astrocytes, co‐cultured with neurons prior to kainate addition, delay neuronal cell death (a). Quantification of western bands (b) involved normalizing to Glyceraldehyde Phosphate Dehydrogenase (GAPDH), which was also used as a sample loading control. MAP2 A+B protein levels are expressed as a % of controls (with and without astrocyte conditioning). Control protein levels are represented by a red line. Significance was derived using students *t*‐test (*p < 0.05, **p<0.01, ***p < 0.001). Immunohistochemistry (c‐d) showing MAP2 primary cortical neuron cultures, co‐cultured with astrocytes (c). Neurodegeneration is observed as lack of MAP2‐positive staining (d) after addition of kainate. SHH‐treated astrocytes delay kainate‐induced neurodegeneration (e). Error bars represent standard error of the mean (SEM) Scale bar = 100 μm. *N* = 3 independent cultures.

Using western blotting, we found that kainic acid (100 μM, 24 h) significantly reduced MAP2 protein levels in primary neuronal cultures (90 ± 5% decrease, *p* = 0.0001, DF = 4, *n* = 3) when compared to vehicle controls. Comparing neurons conditioned with astrocytes on transwells to neurons which had been cultured with transwells and then treated with kainic acid (100 μM), using western blotting, we found that kainic acid also significantly reduced MAP2 protein levels of neurons (84.4 ± 4.6% decrease, *p* = 0.0003, DF = 4, *n* = 3). Neurons which had been conditioned with SAG‐treated astrocytes (1 μM, 4 h) also displayed neuronal death following kainate treatment compared to vehicle control: MAP2 protein levels were 60 ± 12% decreased (*p* < 0.001, DF = 4, *n* = 3) compared to neurons conditioned with untreated astrocytes. However, SHH treatment of astrocytes caused attenuation of neuronal death: comparing neurons conditioned with SHH‐treated astrocytes and then treated with kainic acid to neurons conditioned with untreated astrocytes and then treated with kainic acid, we observed significantly higher levels of MAP2 (24 ± 3% increase, *p* = 0.0235, DF = 4, *n* = 3). These data show that SHH‐sensitive astrocytes reduce kainic acid‐induced neurodegeneration (Fig. 6a and b).

To corroborate western blot data, equivalent experiments were conducted using MAP2 immunohistochemistry (Fig. 6c). Neurons conditioned with untreated astrocytes show drastic MAP2 loss (Fig. 6d). Neuronal cultures conditioned with SHH‐treated astrocytes show more MAP2‐positive cells indicating the protective effect of SHH‐treated astrocytes (Fig. 6e). Kainate also caused noticeable neuronal blebbing indicative of neuronal degeneration (Fig. 6d) which is improved with the addition of SAG (Fig. 6e). Quantification of fluorescence revealed that SHH‐sensitive astrocytes increase MAP2 levels in comparison to neurons conditioned with untreated astrocytes, treated with kainic acid (*p* = 0.04, *n* = 3, Figure S2).

## Discussion

We show that SHH pathway activation causes elongation of astrocytes, along with decreases in two markers of mature astrocytes: GLT‐1 and GFAP. Our results confirm that astrocytes are sensitive to SHH agonists. SHH signalling upstream of Gli‐1 is mediated by p38 MAPK in primary cortical astrocytes (Atkinson *et al*. 2009) and these kinases are documented to have multiple functions, including activation of NFκB signalling pathways. Our results also confirm that SHH signals transform astrocytes and increase proliferation, as previously described (Sirko *et al*. 2013). Since Gli1 but not Gli2 or Gli3 are regulated by SHH agonists, our results suggest that the SHH effects are mediated via Gli1 contrary to previous published observations, which show Gli2‐mediated effects in astrocytes (Yang *et al*. 2012; Chechneva *et al*. 2014). Other research, however, shows that Gli1 up‐regulates Ptch1 target genes in both astrocytomas and medulloblastomas, but Cyclin D2, Plakoglobin, paired box protein Pax‐6, and NKX2.2, only in medulloblastomas. This indicates that SHH signalling is also different between pathologies (Shahi *et al*. 2010).

We show for the first time SHH regulation of GLT‐1 in astrocytes. GLT‐1 is a plasma membrane protein responsible for l‐glutamate homoeostasis in the CNS preventing excitotoxicity during synaptic transmission (Danbolt 2001; Martinez‐Lozada *et al*. 2016). While reduction in GLT‐1 has been associated with neuronal damage (Rothstein *et al*. 1996), other studies have suggested that increasing GLT‐1 after pathology can have adverse effects (Li *et al*. 2014). Loss of the glutamate transporter, GLT‐1 induced by SHH agonists was transient, with SHH pathway activation causing an increase in GLT‐1 transcript levels leading to recovery of GLT‐1 within 24 h. These data show an increased protein turnover of GLT‐1 accounting for the loss by 12 h and that this loss is corrected through increased GLT‐1 transcription. Reduced GLT‐1 following SHH may contribute to excitotoxic neuronal damage, through reduced ability of astrocytes to take up glutamate, or conversely reduced GLT‐1 may result in less astrocyte glutamate release that is dependent on transporter reversal. Since SHH‐treated astrocytes suppress neuronal firing and promote neuroprotection to an excitotoxic stimulus, we do not believe GLT‐1 regulation to be the determinant of the astrocyte SHH effects on neurones. SHH treatment of astrocytes caused a decrease in GFAP expression that was independent of changes in GFAP mRNA levels. Our findings suggest that SHH signalling is a brake to astrogliosis, consistent with the suggestion of Garcia *et al*. (2010) who showed that disrupting SHH produced mild astrogliosis. Astrogliosis, is associated with morphological changes, and up‐regulation of the intermediate filament protein GFAP (Eddleston and Mucke 1993; Pekny and Nilsson 2005) (Middeldorp and Hol 2011), and the current work shows SHH affects these features. Recent reports suggest GFAP reduction, combined with morphological changes indicate a form of astrocyte de‐differentiation (Sirko *et al*. 2013). This de‐differentiation has been demonstrated both *in vitro* (using Transforming growth factor alpha (TGFα)) (Zhou *et al*. 2001; Sharif *et al*. 2007) and *in vivo* using SHH (Sirko *et al*. 2013). This phenomenon is interpreted as the ability of mature astrocytes to transform into multipotent self‐renewing stem cells (Lang *et al*. 2004; Yang *et al*. 2009). Our data show that astrocytes undergo a robust change in phenotype when stimulated with SHH agonists.

As well as characterising some of the phenotypic and biochemical effects of SHH pathway activation on astrocytes, we tested the functional effect of the SHH agonist SAG on astrocytes in regulation of neuronal network activity. We show that neurons become hyperexcitable following SAG administration, in a concentration‐dependent manner and, at higher concentrations, SHH caused neuronal cell death as visualized by MAP2 staining. This suggests that SHH signalling requires tight control to avoid highly detrimental effects to neurons. In astrocyte‐neuron co‐cultures, SHH pathway activation by SAG no longer stimulated neurones, but instead inhibited neuronal firing frequency. We showed that this inhibition was dependent on metabolically active astrocytes since ethylfluoroacetate abolished the inhibitory effect of SHH pathway activation on suppression of neuronal firing in co‐cultures. These results show that the astrocyte‐mediated inhibition of neuronal excitability is not because of sequestration of SHH agonists, but requires a factor, currently not identified, that is dependent on active astrocytes. In support of this notion, recent evidence shows that hedgehog pathway signalling can control the release of d‐serine, glutamate and ATP from cerebellar astrocytes (Okuda *et al*. 2016).

To extend our understanding of the influence of SHH‐treated astrocytes on neurons, we used Alvetex well inserts in neuroprotection assays. Such inserts allow culture of astrocytes and neurons in the same culture vessel without physical contact. Our results show that SHH‐treated astrocytes protect neurons from kainate‐induced cell death as determined by assaying MAP2 loss. These *in vitro* results correlate and extend previous *in vivo* research which has shown that SHH signalling is protective, reducing infarct size in stroke (Chechneva *et al*. 2014) and protecting against kainic acid induced neurodegeneration (Pitter *et al*. 2014). Furthermore, our use of transwells to physically separate astrocytes from neurons without the confounding effect of SAG in the media shows that this protection is mediated through factors secreted from SAG‐treated astrocytes and not direct astrocyte‐neuron interactions or by SAG effects on neurones directly. Our results suggest that the reduction in astrocyte reactivity caused by SHH can directly influence neuronal survival.

As previously discussed, the morphological (shape change) and biochemical (GFAP, GLT‐1) changes conferred by SHH pathway activation are reminiscent of SHH‐induced reduction in astrocyte reactivity (GFAP levels) observed *in vivo*, and we note that this phenotypic change in astrocytes is accompanied by increased neuroprotection. There is a significant evidence base to associate astrocyte phenotype as measured by GFAP levels with neurodegeneration. Multiple reports show reactive astrocytes exacerbate ongoing pathology in various CNS disorders like epilepsy (Zhu *et al*. 2012; Robel *et al*. 2015), motor neuron disease (Diaz‐Amarilla *et al*. 2011) and Alzheimer's disease (AD) (Steele and Robinson 2012). We also note there is contrary evidence suggesting that reactive astrocytes, particularly during glial scar formation, are neuroprotective (Faulkner *et al*. 2004; Sofroniew 2009). Our present findings, together with evidence that astrocytes are enriched in the proteins essential for SHH signalling suggest that neuronal SHH is an important physiological cue for astrocytes in the normal CNS, that mediates astrocyte‐neuron communication to help limit neuronal excitability and confer neuroprotection.

In pathological conditions, where astrocytes become reactive they can themselves secrete SHH (Yang *et al*. 2012; Pitter *et al*. 2014), which may compensate for lost neuronal SHH. SHH is elevated after CNS injury (Amankulor *et al*. 2009). We propose that neuronal SHH acts as a homeostatic signal, providing information about the local microenvironment to astrocytes. Loss of this signal, when neurons are damaged or die, provides a physiological cue, informing astrocytes about the change in the microenvironment and causing astrocytes to become reactive and themselves secrete SHH that can induce astrocyte cell division, reduce reactivity and induce a more protective phenotype.

In summary we show that, *in vitro*, SHH signals through astrocytes to alter their phenotype, suppress neuronal firing and protect neurons from excitotoxic damage. Our findings lead to the hypothesis, supported by other published evidence that SHH is an endogenous neuroprotective signal, which acts through astrocytes.

## Author contribution

C.I.U., W.D.H. and M.R. designed research; W.D.H. and B.J.W., provided analytical tools; C.I.U. and I.S. carried out experiments; C.I.U., B.J.W., W.D.H. and M.R. analysed data; C.I.U., W.D.H. and M.R. wrote the paper.

## Supporting information


**Figure S1.** Morphometric quantification of astrocyte length at 4 hour intervals.
**Figure S2.** Fluorescence quantification of neurons treated with kainate (+Kai) in the presence or absence of astrocytes in the scaffold Alvetex (Alv).
