# Supplementary material for: Sonic hedgehog signalling mediates astrocyte crosstalk with neurons to confer neuroprotection
Source: J Neurochem. 2017 Jun 20;142(3):429–43. doi: 10.1111/jnc.14064 (PMC5575469; doi:10.1111/jnc.14064)
Supplement: Supplementary file 1 — Figure S1. Morphometric quantification of astrocyte length at 4 hour intervals. Figure S2. Fluorescence quantification of neurons treated with kainate (+Kai) in the presence or absence of astrocytes in the scaffold Alvetex (Alv). [file JNC-142-429-s001.pdf]

SHH signalling mediates astrocyte crosstalk with neurons to confer neuroprotection.

Christopher I Ugbode, Imogen Smith, Benjamin J. Whalley, Warren D. Hirst, Marcus Rattray

Supplementary Figure 1

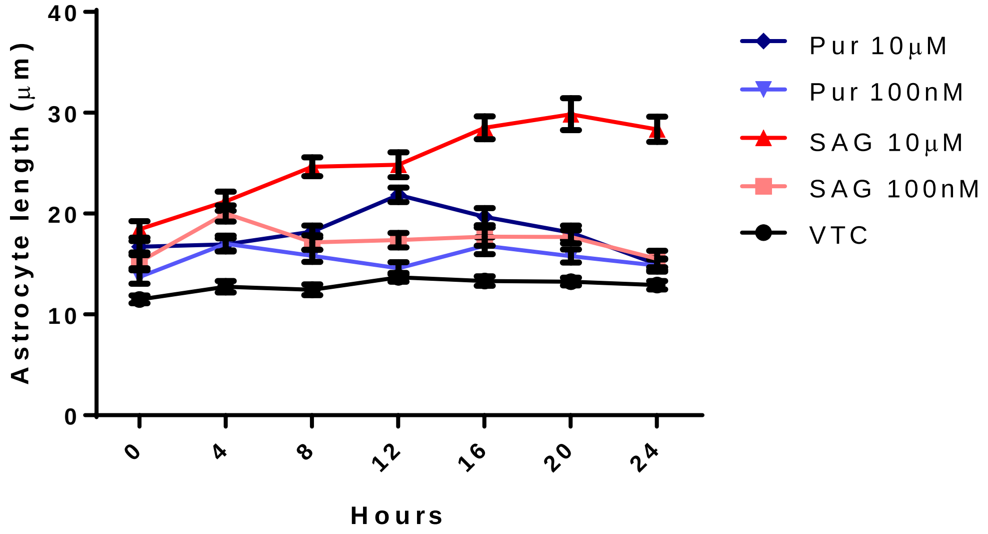

Morphometric quantification of astrocyte length at 4 hour intervals. All agonists increase astrocyte length with smoothened agonist (SAG) at concentrations of 10µM causing sustained elongation. Furthermore, SAG has a more potent effect than Purmorphamine (Pur) at the same concentrations. n = 3 videos from 3 independent cultures analysed per treatment.

Supplementary Figure 2

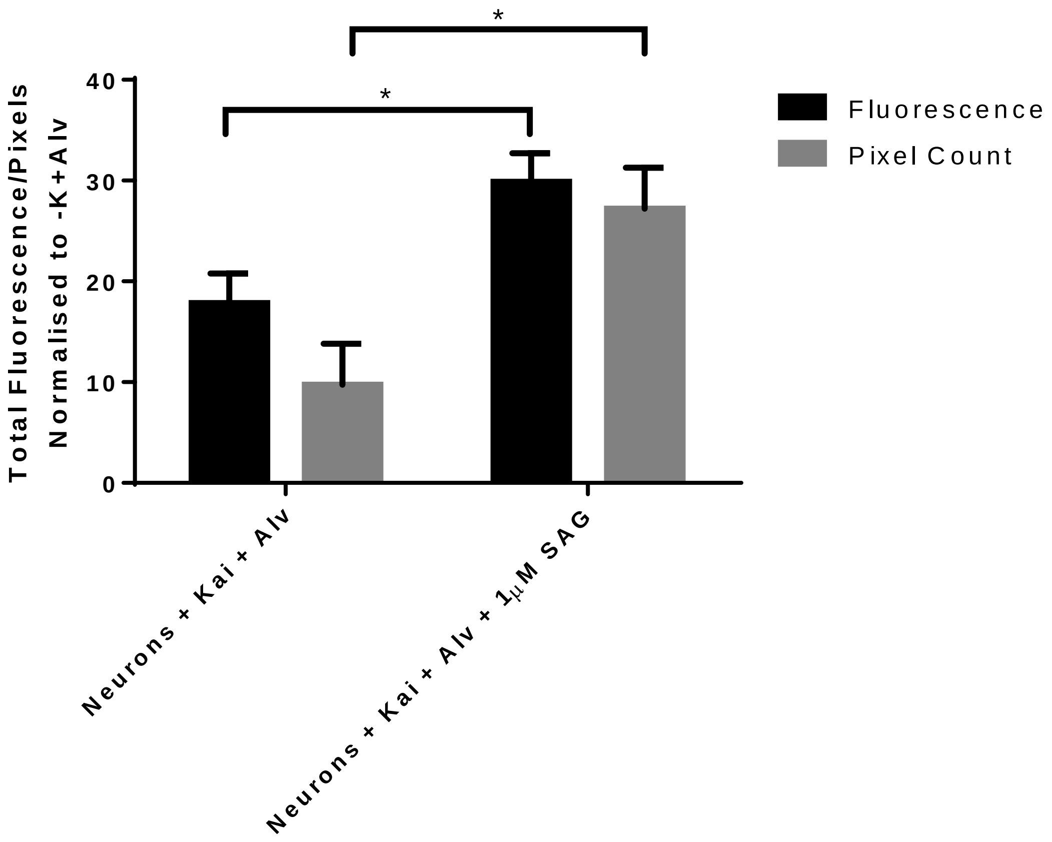

Fluorescence quantification of neurons treated with kainate (+Kai) in the presence or absence of astrocytes in the scaffold Alvetex (Alv). Some astrocytes had been treated with 1µM smoothened agonist (SAG) before co-culture with neurons and addition of 100µM kainate. Neurons co-cultured with astrocytes which had been previously been treated with SAG are better protected from excitotoxic cell death. Total fluorescence and total pixel count are normalised to neurons co-cultured with untreated astrocytes and without addition of kainate. Statistical significance was derived using *Students t-test*. p = <0.05. N=3 independent cultures.
